# Breast metastasis from a pulmonary adenocarcinoma: Case report and review of the literature

**DOI:** 10.3892/ol.2019.10965

**Published:** 2019-10-04

**Authors:** Alessandro Sanguinetti, Francesco Puma, Roberta Lucchini, Stefano Santoprete, Roberto Cirocchi, Alessia Corsi, Roberta Triola, Nicola Avenia

Oncol Lett 5: 328-332, 2013; DOI: 10.3892/ol.2012.995

An interested reader drew to our attention the fact that quite a substantial amount of the text, as well as data featured in Figs. 1–4, which had appeared in the above article had already been published in the following paper [Maounis N, Chorti M, Legaki S, Ellina E, Emmanouilidou A, Demonakou M and Tsiafaki X: Metastasis to the breast from an adenocarcinoma of the lung with extensive micropapillary component: a case report and review of the literature. Diagn Pathol 5: 82, 2010].

An investigation was launched, and the authors were contacted. Dr Sanguinetti responded and acknowledged that a gross error had been made, but was unable to offer an explanation as to how this had occurred. We were unable to contact the other authors. On those grounds, this paper will be retracted from the publication. The Editor deeply regrets any inconvenience that this retraction has caused to the readership of the Journal, and to the authors of the previously published paper.

